# Prevalence of bovine abortion, calf mortality, and bovine viral diarrhea virus (BVDV) persistently infected calves among pastoral, peri-urban, and mixed-crop livestock farms in central and Northwest Ethiopia

**DOI:** 10.1186/s12917-021-02798-w

**Published:** 2021-02-19

**Authors:** Erdachew Yitagesu, Wendi Jackson, Nigatu Kebede, Woutrina Smith, Tsegaw Fentie

**Affiliations:** 1 Debre Birhan Agricultural Research Centre, Debre Birhan, Ethiopia; 2 University of Gondar, College of Veterinary Medicine and Animal Sciences, Gondar, Ethiopia; 3 University of California, School of Veterinary Medicine, One Health Institute, Davis, CA USA; 4 Addis Ababa University, Aklilu Lemma Institute of Pathobiology, Addis Ababa, Ethiopia

**Keywords:** Abortion, BVDV, Calf, Mortality, Ethiopia, Persistent infection

## Abstract

**Background:**

Bovine Viral Diarrhea virus (BVDV) is one of important diseases of cattle worldwide causing economic losses to the cattle industry primarily due to increased premature culling and decreased reproductive performance. The virus can cross the placenta during early pregnancy and result in the birth of persistently infected (PI) calves that are efficient transmitters of BVDV and serving as the primary reservoirs for BVDV. Relatively few studies have focused on understanding BVDV seroprevalence, virus detection, genotyping and its distribution in Africa. Most BVDV research in Ethiopia has involved serologic surveys in adult cattle, rather than the identification of PI calves, despite their role in viral shedding and recurring infections. A cross-sectional study was undertaken in three different livestock production systems of Ethiopia with the objective to estimate the prevalence of bovine abortion, calf mortality, and BVDV persistently infected calves.

**Results:**

Ear notch samples (882) collected from calves in 349 households were tested for BVDV antigen using antigen capture enzyme-linked immunosorbent assay (ACE). All samples tested were negative for BVDV antigen. The overall animal level crude abortion and calf mortality prevalence were 4.0% (95% CI: 2.9–5.2) and 9.2% (95% CI: 7.7–11.0) respectively. The lower BVDV PI prevalence may be due to a lower effective contact rate between cattle reared in small-scale extensive production systems in Ethiopia.

**Conclusions:**

This is the first report of BVDV Ag test in Ethiopia and no PI was detected in calves in the study areas. Since BVDV is a disease of great economic importance, this study finding must be interpreted with care since absence of evidence is not evidence of absence and even a single BVDV infected animal can serve as source of infection and contribute to the persistent spread of the virus. Greater attention needs to be given to screening for PI animals through testing large number of animals and culling positive animals. Hence, future research should focus on regions and production systems with high BVDV seroprevalence followed by antigen ELISA or BVDV real-time PCR to detect persistently infected and acutely viremic animals.

## Background

Bovine viral diarrhea virus (BVDV) is an important pathogen that belongs to the family *Flaviviridae* and genus *Pestivirus*. The flaviviruses are a family of small enveloped viruses with positive-strand RNA genomes of approximately 9.0 to 13 kbp [1]. Two genotypes, BVDV1 and BVDV2, are identified as distinct species within this genus, with further classification as cytopathic (CP) and noncytopathic (NCP) based on in vitro cell culture characteristics and genetic differences [2]. Noncytopathic is the most common naturally occurring biotype, but cytopathogenic effects on cultured cells do not relate to virulence in vivo, since most virulent, i.e. disease causing, strains are of the noncytopathic biotype [3].

Bovine viral diarrhea virus is one of the most economically important diseases of cattle worldwide causing direct economic losses through mortality, morbidity, premature culling, and reproductive losses including reduced conception rates, early embryonic death, abortions, congenital defects, and weak calves. It also causes indirect economic impacts through expenditures on vaccination, individual animal testing, and other control measures [4, 5].

Control programs in many countries of the world depend upon the detection of PI animals, eliminating them and preventing their return into the herd. Recent advances allow better detection of PI animals at an early age, soon after birth and thus improve the possibility of enhanced control [6, 7]. BVDV antigen-ELISA and RT-PCR are the two most reliable and sensitive methods for detecting PI animals, with antigen-ELISA being the most cost-effective for testing large numbers of animals [8]. Two conserved and immunogenic BVDV proteins have been identified as target antigens for ELISA, the non-structural protein NS3 (p80) and the envelop glycoprotein Erns (E0). NS3 is highly conserved among all BVDV strains but its concentration can be low outside of infected cells. Erns is also highly conserved and remains detectable in ear-notch samples from young animals even in the presence of colostral antibodies [9]. Acute, transiently infected (TI) animals can be difficult to detect relative to PI animals because the virus is only detectable in the blood and occasionally in ear-notch samples for approximately 3 weeks and infected animals may display only mild clinical signs or none at all [10, 11]. In order to differentiate between PI and TI animals, it is recommended to sample twice at three-week intervals to confirm infection status [8].

Although BVDV infects various domestic and wild ruminants, cattle are the natural host developing the clinical disease in the ranges of inapparent to severe, with a high mortality rate and potential involvement of one or more organ systems [6]. The birth of persistently infected (PI) calves with BVDV resulting from in utero fetal exposure to the virus is extremely important in the perpetuation of the virus in an infected herd and in the transmission of BVDV to susceptible herds through the introduction of a PI calf. This virus establishes persistent infection by invading the fetus early in its intrauterine development, thus establishing infection characterized by immunotolerance specific for the persisting viral strain. When the NCP biotype of BVDV infects a seronegative dam during the first trimester of gestation, the immature immune system of the fetus is not able to develop a sufficient immune response, and therefore becomes immunotolerant to the virus resulting in a PI calf. Animals infected during this critical phase of gestation (approximately from day 40 to day 120 of gestation) may develop and be born normally but remain persistently infected for their entire life shedding high levels virus into the environment [12]. Calves born PI will often appear normal at birth, but may be immunosuppressed and frequently die in the first year of life due to increased susceptibility to infectious diseases like pneumonia [6].

Bovine viral diarrhea virus is distributed globally, and its antibody (Ab) prevalence in cattle varies among countries. In many countries where cattle are reared, BVDV’s distribution and its impact upon the livestock industry is known [5]. Seroprevalence values pooled from 73 countries range from 46.2 to 48.7% at the animal level and from 66 to 67% at the herd level. Most of BVDV prevalence assessments are conducted in Europe and North America, and the least number of reports are from Africa [13]. In contrast, relatively few studies have focused on understanding BVDV Ab prevalence, virus antigen detection, and genotyping and its distribution in Africa. Research by Elhassan et al. [14] reported 76% BVDV Ab prevalence in two regions of Sudan and 84.3% seroprevalence in dams with a history of abortion. Later, Saeed et al. [15] found BVDV1 antigen in 10.7% of lung tissue samples collected from pneumonic dairy cattle at a slaughter house in Sudan. BVDV1 Ab prevalence in South African dairy and beef cattle has been reported to range from 60 to 66% [16]. Approximately, 9.7% of fetal losses from dairy cattle in Nakuru, Kenya were attributed to BVDV infections based on seroconversion in a prospective study and a total of 79% dairy cattle were seropositive for BVDV in the same study [17]. Results from these serologic surveys indicate that exposure to BVDV is relatively high among cattle in various parts of Africa.

Cattles are vital to Ethiopians’ livelihoods and contribute significantly to the national economy, yet their productivity remains low relative to other East African countries like Kenya, due to endemic disease, reproductive losses, poor management, and mortality [18, 19]. The association between BVDV and reproductive losses and calf mortality has been documented in many countries but little is known about its distribution in Ethiopia. Recently, BVDV seroprevalence in central and southwestern Ethiopia was reported between 11.46 and 32.6% at the animal level and 69.8% at the herd level [20, 21]. Approximately, 35% of dairy cattle that aborted were seropositive for BVDV, and 28% of weak calves examined were also seropositive [21]. Since vaccination against BVDV is not practiced in Ethiopia, results from seroprevalence studies represent antibodies produced from field exposure or maternal antibody transfer (young calves) rather than vaccination.

While the seroprevalence of BVDV is well documented in literature, only a small subset of studies have evaluated seroprevalence relative to clinical disease and even fewer have focused on the prevalence of PI animals. The worldwide pooled PI prevalences at the animal level range from low (≤0.8% Europe, North America, Australia), medium (> 0.8 to 1.6% East Asia) to high (> 1.6% West Asia) based on a meta-analysis study [13]. The prevalence of PI cattle in South African feedlots was reported at 2.9% [22]. Epidemiological investigations have shown that demographic factors such as herd size and density are significant predictors for the prevalence of infection in populations where BVDV is endemic [7].

The higher BVDV seroprevalence reported in Ethiopia in the past decade, may point towards the increasing importance of this pathogen in cattle. Livestock development goals in Ethiopia prioritize improving cattle productivity, making it imperative to understand the prevalence and distribution of BVDV infection, to inform prevention and control measures. Prior BVDV research in Ethiopia has focused on adult cattle and Ab prevalence, and to our knowledge, not on the identification of PI calves, despite their significant role in viral shedding and recurring infections. Therefore, the objectives of this study were to estimate the prevalence of bovine abortion, calf mortality, and BVDV antigen (Ag) positive calves, and evaluate potential risk factors across three different animal production systems in Ethiopia.

## Results

**Fig. 1:**
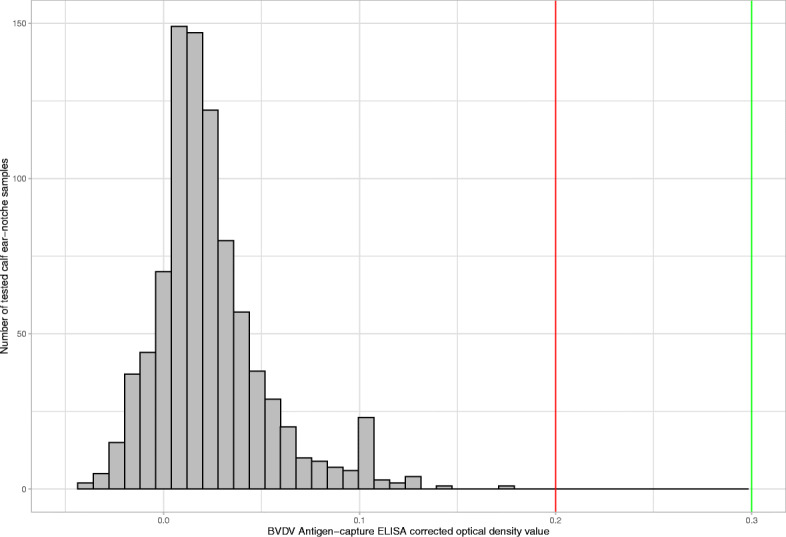
Histogram of ear notch and serum sample AC-ELISA BVDV corrected optical density test results. Note: the area between the green and red vertical line (0.200 < corrected OP value <= 0.300) represents values that are suspect for BVDV antigen, values less than or equal to the green line (0.200) are negative and values greater than the red line (0.300) are considered positive

**Fig. 2:**
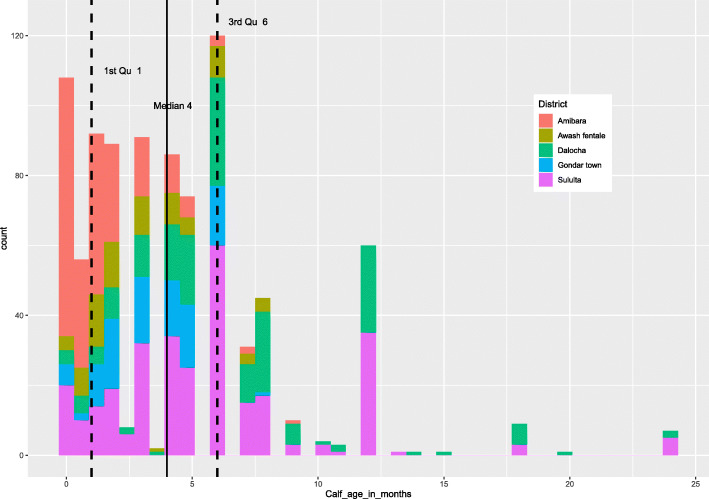
Age distribution of sampled calves from each district (*n* = 882)

Descriptive information about the sampled animals is presented in Table 1. A total of 882 ear notch samples collected from 349 households were tested for BVDV antigen from the five districts (Table 1). All 882 samples tested negative for BVDV antigen. The corrected optical density values of BVDV Ag tested samples (882) were in the negative range (− 0.01 to 0.07 corrected OD) based on the manufacturer’s calculation (Fig. 1). The majority of samples were collected from calves aged 1 to 6 months with the median age being 4 months. Most of the sampled calves from Amibara and Awash Fentale district were younger than 4 months of age. Calves and adult cattle sampled from Sululta and Dalocha district ranged in age from 1 day old up to 24 months (Fig. 2).

**Table 1:** Descriptive information about herd size, number of kebeles and samples collected per district in central and northwest Ethiopia

Districts	Kebeles selected within districts	Number of HHs sampled	Cattle herd size per HH				Number of sampled animals
			Mean	Median	Min	Max	
Sululta	3	110	13	10	2	180	301
Dalocha	3	134	5	5	1	16	183
Amibara	1	42	30	25	3	70	205
Awash Fentale	2	31	19	16	4	45	81
Gondar town	10^a^	32	13	7.5	1	124	112
Total	19	349	16	**7**	–	–	882

^a^Number of kebeles selected from Gondar town was higher since only a few dairy farms are dispersed in each kebele, *HH* household, *min* minimum, *max* maximum

The overall calf crude mortality prevalence in the five districts was 9.2% (95% CI: 7.7–11.0). Of the 1196 total calves born across the five districts in the past year prior to the survey, 111 (9.2%) calves had died. The highest crude mortality prevalence of 32.4% (95% CI: 25.5–40.2) was recorded in Awash Fentale, and lowest, 1.9% (95% CI: 0.48–7.3) in Gondar town (Table 2).

**Table 2:** Calf mortality prevalence by district based on calf births and deaths in the previous year

Districts	Number of calves born	Number of calf deaths	Prevalence (%)	SE	95% CI
Sululta	431	30	6.9	1.2	4.9–9.7
Dalocha	170	7	4.1	1.5	1.9–8.3
Amibara	336	22	6.5	1.3	4.3–9.7
Awash Fentale	154	50	32.4	3.7	25.5–40.2
Gondar town	105	2	1.9	1.3	0.4–7.3
Total	1196	111	9.2	0.01	7.7–11.0

*SE* Standard error, *CI* Confidence interval

The overall abortion prevalence in the five districts was 4.0% (95% CI: 2.9–5.2). Of the 1246 total cows pregnant in the year prior to the survey, 50 (4.0%) cows had aborted. The highest abortion prevalence of 8.8% (95% CI: 5.4–14.2) was recorded in Awash Fentale, and the lowest, 1.1% (95% CI: 0.2–4.5) in Dalocha (Table 3).

**Table 3:** Abortion prevalence by district based on cow pregnancies and abortions in the previous year

Districts	Number of pregnancies	Number of Abortions	Prevalence (%)	SE	95% CI
Sululta	442	11	2.4	0.7	1.3–4.4
Dalocha	172	2	1.1	0.8	0.2–4.5
Amibara	355	19	5.3	1.1	3.4–8.2
Awash Fentale	169	15	8.8	2.1	5.4–14.2
Gondar town	108	3	2.7	1.5	0.9–8.2
Total	1246	50	4.0	0.5	2.9–5.2

*SE* Standard error, *CI* Confidence interval

## Discussion

This study on the prevalence of BVDV PI calves based on antigen ELISA is the first report of its kind in Ethiopia. In the present study, BVDV Ag was not detected in ear-notch samples from the large number of sampled calves. This may indicate a low prevalence of PI and transiently infected calves among the districts and kebeles in central and northwest Ethiopia enrolled during the sampling period in this cross-sectional study. In Ethiopia recent studies indicated that BVDV seroprevalence is in the range of 11.46 to 32.6% [20, 21]. Bovine virus diarrhea antigen prevalence and BVDV Ab prevalence are directly proportional, i.e. presence of a few PI calves in a herd results in a higher rate of virus exposure and transient infections of most animals. Thus, the level of seroprevalence is indicative of the level of PI animals in a herd since TI animals are less efficient at transmitting [23, 24]. Houe and Meyling [24] and Houe [23] studied BVDV Ab prevalence of a small number of cattle from a herd to predict presence or absence of PI animals in the herd. They found 87% BVDV Ab prevalence in the presence of a PI calf in the herd and 43% BVDV Ab prevalence in the absence of a PI calf in the herd, indicating that TI animals still have a role in transmission within herds. In the same study, BVDV Ab prevalence increased to 97%, 6 months after the introduction of a PI calf into a seronegative herd. The probability of detecting BVDV seropositive animals in herds with PI animals ranges from 0.72–0.97 and 0–0.05 from herds having no PI animals. Similarly, in Switzerland, BVDV Ab prevalence was reported at 57.6% in herds in which the prevalence of PI calves was 0.64% [25]. Based on this BVDV PI prevalence prediction from BVDV antibody seroprevalece, our result is consistent with antibody seroprevalence studies reported in Ethiopia. Our findings are also consistent with the 0% BVDV antigen prevalence detected in Cameroon, although in the same study, the BVDV Ab based prevalence was 30% at the individual animal-level [26]. In contrast to the findings in this study, a high prevalence of BVDV Ag prevalence was reported at 10.7% by Saeed et al. [15] in Sudan and 4.7% by Kabongo and Vuuren [27], in South Africa. However, they sampled animal specimens from clinical cases and this increases the detection rate of PI calves. Worldwide pooled PI prevalence at the animal level ranges from 0.8 to 1.6% [13].

Although BVDV Ab prevalence was not a component of the present study, the relatively low seroprevalence values from previously reported studies conducted in Ethiopia [20, 21] may provide justification for why PI calves were not detected in this study. The smaller herd size, extensive management, and low stocking density found in communal grazing areas in Ethiopia may lower the effective contact rate required for BVDV transmission compared to large scale intensive dairy systems in Europe and USA [28]. To increase the probability of detecting PI calves, sampling high-risk herds with a historically high BVDV Ab prevalence and/or history of abortion, diarrhea, or low conception rates would be recommended, but this was not the objective of the current study. In the present study, the prevalence of calf mortality and abortion was relatively small across the three production systems (Tables 2 and 3). Findings from other studies indicate that the prevalence of PI cattle is highest in dairy production systems, in non-vaccinated herds, in animals with clinical signs, and in herds not enrolled in control/prevention programs [13]. A stochastic BVDV spread model showed that herd size is an important factor in maintaining BVDV infection. The model predicts that dairy farms with a herd size of 50 and 100 animals can clear BVDV infection by between 4 and 9 years, respectively, but a farm with a herd size of 400 would take more than 10 years to become BVDV-free [28]. In the current study, the herd size per household was relatively small (mean herd size = 16 cattle) (Table 1). In Ethiopia where the majority of cattle are reared in extensive farms there may be reduced risk for transmission of BVDV or a shorter duration of PI animals in the herd due to a shortened lifespan.

In this study, the BVDV Erns antigen was targeted for detection because it is conserved in the structural region of the virus and is the most appropriate protein for detection in ear notch samples from calves < 6 months of age, when maternal antibodies are still present. However, Gripshover et al. [29] reported there was an atypical mutation in the portion of the genome coding for the Erns glycoprotein AU501 BVDV isolate in North America and perhaps this mutation may also circulate in Ethiopian cattle. Genotyping BVDV virus strains could provide important information on the epidemiology of the virus present in different regions of Ethiopia. A BVDV quantitative PCR assay would also be a valuable tool to use in conjunction with antigen-based assays to confirm BVDV infection status.

In the present study, the estimated average prevalence of abortion and crude calf mortality across the three farming production systems was 4 and 9.2% respectively. The calf crude mortality prevalence (9.2%) reported in this study agreed with the calf morbidity prevalence of 9.3% reported by Megersa et al. [30] and 11.6% reported by Romha, [31] in different livestock production systems of Ethiopia. Similarly, Otte and Chilonda [32] reviewed and reported mortality prevalence ranging from 20.7 to 22.3% in mixed systems of different agro-ecological zones of sub-Saharan African countries. However, calf mortality in the present study was considerably lower than most of the previous reports in different parts of Ethiopia [33]. Wudu et al. [33] reported 22% and Ferede [34] reported 17.9% crude mortality rate. The current abortion prevalence is lower than 5.7% abortion prevalence in central Ethiopia [35]. Reproductive disorders are a major concern in Ethiopia and abortion is the disorder most frequently reported. Morbidity from reproductive disorders account for 24.6% of clinical disease in dairy cattle [36]. Reproductive disorders can lead to reduced reproductive performance by decreasing fertility and increasing calving interval as a result of abortion, retained placenta, metritis, among others [35].

A limitation of this study was that only calves 6 months of age and younger, with a few exceptions, were enrolled and sampled since it was part of a larger multi-year project focused on young stock mortality in Ethiopia. Although, PI calves are implicated as the most important BVDV transmitters, their increased susceptibility to other calfhood diseases like pneumonia and diarrhea, may contribute to an early death in the neonatal period, reducing their detection in the present study and their ability to transmit virus. On the other hand, there may be some PI calves that survive into adulthood which would have been missed in this study. Another limitation included relying on farmer recall in determining abortion prevalence which may have introduced bias. Depending on the location of the dam at the time of abortion, recall of an aborted fetus may have been underreported if it was unobserved by the farmer. However, the broad approach taken to cross-sectional sampling in this study enabled a comprehensive investigation of PI calves across three different productions systems in geographically diverse regions of Ethiopia. Another approach to consider in future work, which was not possible in this study due to limitations on implementing BVDV real-time PCR, would be to PCR test pooled samples from farms in order to reduce the cost of screening individual animals followed by individual animal testing for confirmation from positive pooled samples. Testing for BVDV antigen using pooled ear-notch samples is not recommended by the manufacturer of the BVDV ACE kits.

## Conclusions

In the current study, we did not detect BVDV transiently or persistently infected calves using the ACE assay on ear-notch samples in the selected study areas. The 0% antigen prevalence and previously reported BVDV Ab prevalence in Ethiopia are considerably lower than other BVDV endemic countries. Since BVDV is a disease of great economic importance, this study finding must be interpreted with care since absence of evidence is not evidence of absence, and even a single BVDV infected animal can serve as source of infection and contribute to the persistent spread of the virus within a farm and to different areas. To effectively overcome this problem, greater attention needs to be given to screening for PI animals through testing large number of animals and culling positive animals. BVDV serologic surveys followed by ACE or BVDV real-time PCR are recommended to identify PI and acutely viremic cattle of all ages in dairy farms at the national level and systematic epidemiologic investigation and genetic typing of BVDV in Ethiopia will be useful for studies on vaccine and control strategies. Findings from the household questionnaire indicate that abortion and calf mortality are a significant problem to smallholder farmers, especially pastoralists. Future work should include prospective sampling to better associate clinical disease in dams and calves with identification of infectious pathogens and/or management risk factors that may be responsible for abortion and calf mortality.

## Methods

### Description of the study areas

**Fig. 3:**
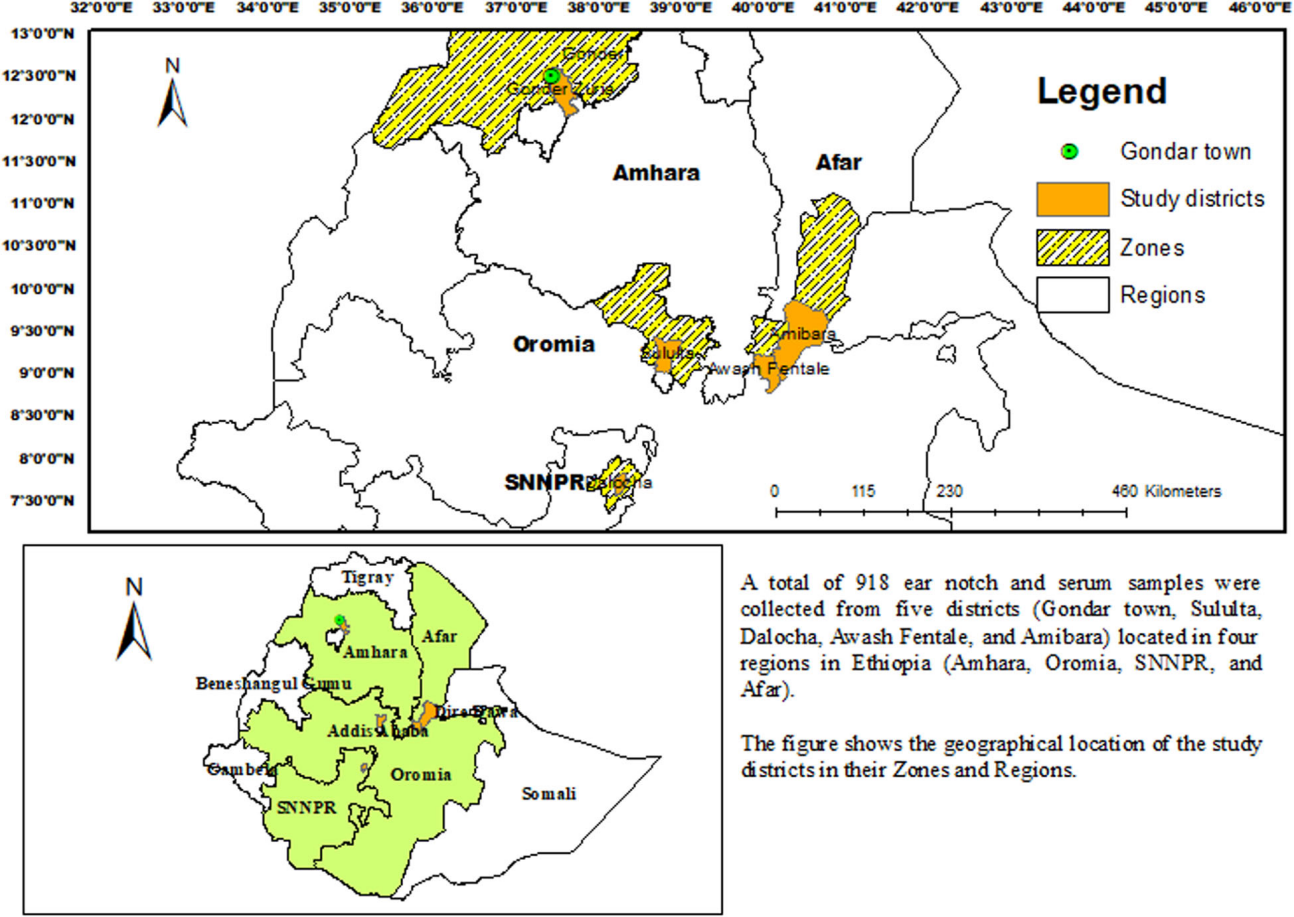
Map of Ethiopia showing the regions where the study zones and districts are located. (The image depicted in this figure was created by the authors)

The study was conducted in five districts selected across four regions of Ethiopia representing peri-urban dairy farms (Sululta, Gondar town), mixed-crop livestock farms (Dalocha), and pastoral herds (Awash Fentale and Amibara). The selected districts included Gondar town (Amhara region), Sululta (Oromia region), Dalocha (South Nations, Nationalities and Peoples Region) and Amibara and Awash Fentale (Afar region). Sululta, Amibara, Awash Fentale, and Dalocha are located in central Ethiopia while Gondar town is in northwest Ethiopia. The location of each study district within its respective region is shown in Fig. 3. A Map (Fig. 3) was developed with ArcGIS 10.7.1 (ESRI Inc., Redlands, CA, USA) to show the study area.

### Study population and sampling method

The study population consisted of Zebu, Holstein Friesian, and crossbred cattle in central and northwest Ethiopia. Animal husbandry in the crop-livestock production system is characterized by a smallholder extensive management system. Cattle and sheep are the predominant animal species kept integrated with crop production. The pastoral production system of the Afar regions is characterized by seasonal mobility of livestock in the search of pasture and water. Cattle are the predominant animal species followed by goats, camels, and sheep. The market-oriented dairy production system is mainly located in urban and peri-urban areas of Addis Ababa, Oromia, and Amhara Regions. Dairy cattle of improved breeds are the predominant animals kept by smallholder and medium scale farmers selected mainly for milk production.

Six kebeles, three each from Sululta and Dalocha, two kebeles from Awash Fentale and one kebele from Amibara district were included in the study. Kebele is the smallest administrative unit in Ethiopia, similar to a neighborhood. Animal health services at the kebele-level consist of a veterinarian based at the kebele and Woreda Office of Agriculture (Woreda are an administrative district comprised of several kebeles), who provide livestock extension services (vaccination, deworming, clinical cases treatment etc.) in the kebele. From each kebele, households having at least one calf less than or equal to 6 months of age were identified for enrolment with the help of an extension veterinarian from each kebele. On rare occasions, calves greater than 6 months of age were enrolled and sampled, when a kebele had only a small number of eligible calves <= 6 months of age available.

### Study design and sample size determination

This cross-sectional study used a multi-stage cluster sampling method in each study district. All household enrolment and sampling took place between September 2018 and December 2019. Sample size was calculated using an equation based on estimating proportions. However, since BVD infection has been found to have a high intra-cluster correlation coefficient (ρ or rho) = 0.31 (0.21 to 0.45) in dairy herds [37], and considering kebele (village) as a cluster in our study, the sample size was calculated using the formula described by Bennett et al. [38].

1 $$ SE=\sqrt{\frac{pqD}{n}}=\sqrt{\frac{pqD}{cb}} $$

Where p is a priori *estimate* of the proportion, q = l – p, n is the required sample size, c is the number of clusters (kebeles), and b is the number of samples from each cluster (kebele). Sampling 100 animals per cluster (kebele) with a disease prevalence estimated at 2.9% [39] and 9 clusters (kebeles) resulted in an estimate of the standard error (1) or precision of 0.026.

2 $$ \uprho =\frac{\mathrm{with}\ \mathrm{in}\ \mathrm{herd}\ \mathrm{variation}}{\ \mathrm{total}\ \mathrm{variation}} $$

3 $$ D=1+\left(b-1\right)\uprho $$

*Roh* (*ρ*) describes the rate of homogeneity. A *Roh* (*ρ*) of 0.2 [37] was used in relation to sampling 100 animals per cluster (kebele), with a design effect (D) equal to 20.8 (3).

4 $$ c=\frac{pqD}{bSE{.}^2} $$

Sampling 100 animals per cluster (kebele) with an expected BVDV persistent infection prevalence of 2.9% [22] in South Africa and a desired precision of 0.025% resulted in 9 clusters (4) and a total sample size of around 900 animals for this study. The clusters and the total sample size were more or less equally distributed across the study districts and a total of 882 ear notch samples were collected and tested using the BVDV Antigen-Capture Enzyme Linked Immunosorbent Assay (ACE) assay.

### Survey questionnaire and sample collection

A household head or family member having knowledge about the herd and management was interviewed in order to collect household and animal-related demographic data, risk factors, abortion, and calf mortality data using a semi-structured questionnaire. Physical examinations were performed on each calf, and the eye, nasal, ear, body condition, and fecal score were recorded using standardized criteria. Body temperature and the presence/absence of a cough were recorded onto an individual calf record sheet. All calves <= 6 months of age in each household underwent ear notch sample collection for BVDV Ag testing.

Ear notch biopsy was conducted according to the method outlined in Cornish et al. [40]. An ear notch measuring approximately 2–3 mm in diameter was obtained from the dorsal pinna margin of each calf using a small swine ear notcher. Following sample collection, cotton was applied to the ear for 30 s for hemostasis and the ear notcher was rinsed with water and placed in a bottle of 10% bleach solution to disinfect. Prior to use again, the ear notcher was rinsed again with water to remove disinfectant. Ten percent (10%) bleach solution is recommended as a disinfectant for ear-notch tools by the Wisconsin Veterinary Diagnostic Laboratory [41] and widely used in USA. Ear notch biopsy tissue was placed in a cryovial, stored in a cooler for transport to the laboratory, and frozen at ≤ − 20 °C until testing.

### BVDV antigen-capture enzyme linked Immunosorbent assay

Detection of BVDV Ag in skin (ear notch) specimen was performed using the manufacturer’s instructions outlined in the test-kit package insert (BVDV Ag/Serum Plus, IDEXX, USA). This kit is designed to detect BVDV Erns antigen in serum, plasma, whole blood, and ear notch tissue in cattle. The test kit has diagnostic specificity of 100% (95% CI: 95.69–100) and sensitivity 100% (95% CI: 90.50–100) [8]. A micro titration format was configured by immobilizing specific monoclonal antibodies for BVDV (Erns) on the plates that would then bind to BVDV antigen in the sample. After incubation (2 h at 37 °C), of the test sample in the plate, captured BVDV antigen was detected by specific antibodies and horseradish-peroxidase conjugate. Next, an unbound conjugate was washed away and a substrate/chromogen solution was added. In the presence of enzyme, substrate was converted into a product that reacts with the chromogen to generate a blue colour. Upon the addition of the stop solution, a yellow colour was generated. The absorbance was measured using a spectrophotometer at 450 nm wavelength. The corrected OD value of the samples were calculated by using the absorbance obtained with the test sample and corrected for the absorbance of the negative control. The test protocol included adding a positive and negative control to each plate for every run of the assay.

$$ COD\  sample=S-N= Sample\ A\ (450)- NC\  mean $$

Note: COD sample – corrected optical density of sample, NC mean – average optical density of two test readings of the negative control from the plate. S - N sample ≤ 0.200 was recorded as a negative result, S - N sample > 0.300 was recorded as a positive result and 0.200 ≤ S - N sample < 0.300 was considered as a suspect result.

## Data Availability

The survey materials and data collected and analysed in this study are available from the corresponding author upon request.

## Abbreviations

ACE: Antigen Capture Enzyme-Linked Immunosorbent Assay
BVDV: Bovine Viral Diarrhea Virus
ELISA: Enzyme-Linked Immunosorbent Assay
Kbp: Kilo base pair
Ab: Antibody
PI: Persistently Infected
RNA: Ribonucleic Acid
PCR: Polymerase Chain Reaction
